# Inhibition of lipopolysaccharide-induced NF-κB maintains osteogenesis of dental pulp and periodontal ligament stem cells

**DOI:** 10.1590/1807-3107bor-2024.vol38.0037

**Published:** 2024-05-13

**Authors:** Ferry SANDRA, Janti SUDIONO, Angliana CHOUW, Maria CELINNA, Nurrani Mustika DEWI, Melanie Sadono DJAMIL

**Affiliations:** a Universitas Trisakti, Faculty of Dentistry, Division of Oral Biology, Department of Biochemistry and Molecular Biology, Jakarta Barat, Jakarta, Indonesia.; b Universitas Trisakti, Faculty of Dentistry, Division of Oral Biology, Department of Oral Pathology, Jakarta Barat, Jakarta, Indonesia.; c PT Prodia StemCell Indonesia, Jakarta Pusat, Jakarta, Indonesia.; d The Prodia Education and Research Institute, Jakarta Pusat, Jakarta, Indonesia.

**Keywords:** Stem Cells, Dental Pulp, Periodontal Ligament, Lipopolysaccharides, NF-kappa B

## Abstract

Dental pulp stem cells (DPSCs) and periodontal ligament stem cells (PDLSCs) can differentiate into osteoblasts, indicating that both are potential candidates for bone tissue engineering. Osteogenesis is influenced by many environmental factors, one of which is lipopolysaccharide (LPS). LPS-induced NF-κB activity affects the osteogenic potencies of different types of MSCs differently. This study evaluated the effect of LPS-induced NF-κB activity and its inhibition in DPSCs and PDLSCs. DPSCs and PDLSCs were cultured in an osteogenic medium, pretreated with/without NF-κB inhibitor Bay 11-7082, and treated with/without LPS. Alizarin red staining was performed to assess bone nodule formation, which was observed under an inverted light microscope. NF-κB and alkaline phosphatase (ALP) activities were measured to examine the effect of Bay 11-7082 pretreatment and LPS supplementation on osteogenic differentiation of DPSCs and PDLSCs. LPS significantly induced NF-κB activity (p = 0.000) and reduced ALP activity (p = 0.000), which inhibited bone nodule formation in DPSCs and PDLSCs. Bay 11-7082 inhibited LPS-induced NF-κB activity, and partially maintained ALP activity and osteogenic potency of LPS-supplemented DPSCs and PDLSCs. Thus, inhibition of LPS-induced NF-κB activity can maintain the osteogenic potency of DPSCs and PDLSCs.

## Introduction

Several studies have reported that mesenchymal stem cells (MSCs) have potential uses in tissue engineering and regenerative medicine,^1-3^ including the field of dentistry.^4^ Dental pulp stem cells (DPSCs) and periodontal ligament stem cells (PDLSCs) are oral tissue-derived stem cells that possess the properties of MSCs.^4-6^ Under specific culture conditions, DPSCs and PDLSCs can be differentiated into mesenchymal lineages, including osteoblasts.^7-9^ DPSCs and PDLSCs have higher growth potential compared with bone marrow mesenchymal stem cells (BMMSCs),^10^ and possess immunomodulatory activity.^2,3,11^ Hence, DPSCs and PDLSCs are potential candidates for bone tissue engineering and regeneration applications, such as alveolar bone repair.^4^

The process of osteogenesis is influenced by several environmental factors, including inflammatory factors produced by bacteria.^12,13^ Lipopolysaccharide (LPS) is the most common inflammatory factor, which is continuously liberated from Gram-negative bacteria colonizing the periodontal tissues, and can cause inflammatory diseases, such as periodontitis.^14^ LPS activates the nuclear factor kappa-light-chain-enhancer of activated B cells (NF-κB) signaling pathway and induces inflammatory responses.^15,16^ Several studies have reported that LPS-induced NF-κB activity in PDLSCs can be inhibited, enabling undisrupted osteogenesis.^12,13^ However, in other types of MSCs, such as BMMSCs, LPS induces NF-κB activity, but does not alter osteogenic differentiation.^12^ In addition, in adipose-derived mesenchymal stem cells (AdMSCs), LPS induced NF-κB activity and stimulated osteogenic differentiation.^17^ Therefore, NF-κB inhibition affects the osteogenic potency of different types of MSCs differently. The aim this study was to evaluate the effect of LPS-induced NF-κB activity, and its inhibition using a specific inhibitor, Bay 11-7082, in DPSCs and PDLSCs.

## Methodology

### Cells Thawing and Culture

Cryopreserved passage five DPSCs and PDLSCs reported in previous research^6,11^ were thawed and cultured in MesenCult MSC Basal Medium (StemCell Technologies, Vancouver, Canada) supplemented with MesenCult MSC Stimulatory Supplement (StemCell Technologies), 200 U/mL penicillin, 200 µg/mL streptomycin, and 0.5 µg/mL amphotericin (Gibco, Grand Island, NY, USA). DPSCs and PDLSCs were harvested after reaching confluency and used in this study. This study was conducted in accordance with the Declaration of Helsinki. Approval was obtained from the Ethics Committee of Faculty of Dentistry Universitas Trisakti, Indonesia (No. #167/KE/FKG/11/2014). Written informed consent was obtained for the collection of human samples.

### Flow Cytometric Analysis

Flow cytometric analysis was conducted using a BD Stemflow hMSC Analysis Kit (BD Biosciences, Franklin Lakes, USA) to confirm whether DPSCs and PDLSCs had MSC markers as previously described.^11^ DPSCs (1 × 10^7^ cells) and PDLSCs (1 × 10^7^ cells) were incubated with/without marker-specific antibodies as well as their isotypes for positive (CD90, CD105, and CD73) and negative (CD45, CD34, CD11b, CD19, and HLA-DR) markers. FACSCanto II flow cytometer (BD Biosciences) was used to analyze labeled DPSCs and PDLSCs using the FACSDiva software (BD Biosciences). The characteristics of DPSCs and PDLSCs were confirmed using the minimal surface marker criteria for defining MSCs, proposed by the International Society for Cellular Therapy (ISCT).^18^

### *In vitro* Osteogenic Functional Assay

*In vitro* osteogenic functional assay was performed as previously described.^6^ DPSCs (8 × 10^4^ cells) and PDLSCs (8 × 10^4^ cells) were cultured using osteogenic medium containing 10 mM β-glycerophosphate (Sigma-Aldrich, St. Louis, USA), 100 nM dexamethasone (Sigma-Aldrich), and 50 µg/mL L-ascorbic acid (Sigma-Aldrich) on a 6-well plate. DPSCs and PDLSCs were pretreated with/without 100 µM NF-κB inhibitor Bay 11-7082 (Sigma-Aldrich) for 30 min and supplemented with/without 10 µg/mL *Porphyromonas gingivalis* LPS (Wako, Osaka, Japan) for 1, 2, or 3 weeks. After removing the medium, the plates were washed twice with PBS and fixed for 2 min in 4% paraformaldehyde (Wako) in phosphate buffer solution (PBS). This was followed by treatment with glycerol (Bio-Rad, Hercules, USA) at room temperature for 5 min. The cells were washed thrice with distilled water after removal of the fixative. The cells were then stained with 2% alizarin red solution (Sigma-Aldrich) for 20 min. After removing the alizarin red stain, the plates were washed thrice with distilled water. The cells were finally observed and documented under an inverted light microscope (Zeiss, Jena, Germany). The experiment was performed twice in triplicate.

### NF-κB Activity Assay

After pretreatment with Bay 11-7082 for 30 min and LPS supplementation for three weeks, NF-κB activity in DPSCs (2 × 10^6^ cells) and PDLSCs (2 × 10^6^ cells) was determined using NF-κB p65 Transcription Factor Assay Kit (Abcam, Cambridge, UK) in accordance with the manufacturer’s protocol. Nuclear extraction of the treated DPSCs and PDLSCs was performed using the Nuclear Extraction Kit (Abcam) in accordance with the manufacturer’s instructions, before determining NF-κB activity. The nuclear extracts containing NF-κB were loaded into 96-well plates containing dsDNA with NF-κB response element sequence, followed by the sequential addition of rabbit anti-NF-κB primary antibody and HRP-linked goat antirabbit IgG secondary antibody. Results were measured at OD_450_ nm using a spectrophotometer (Bio-Rad). The experiment was performed twice in triplicate.

### Alkaline Phosphatase (ALP) Activity Assay

Following pretreatment with Bay 11-7082 for 30 min and LPS supplementation with/without Bay 11-7082 for three weeks, ALP activity in DPSCs and PDLSCs was evaluated using the colorimetric Alkaline Phosphatase Assay Kit (Abcam) in accordance with the manufacturer’s protocol. Briefly, homogenized DPSCs or PDLSCs (1 × 10^5^ cells) and p-nitrophenyl phosphate (pNPP) were loaded into 96-well plates. The plates were incubated in the dark. This was followed by the addition of the stopping solution, and measurement at OD_405_ nm using a spectrophotometer (Bio-Rad). The activity of ALP (U/L) was calculated. The experiment was performed twice in triplicate.

### Statistical Analysis

IBM SPSS Statistics version 26.0 (SPSS IBM, Armonk, USA) was used to conduct the statistical analyses. The Shapiro–Wilk test was used as a normality test. Comparison of NF-κB and ALP activities of DPSCs and PDLSCs in different treatment groups was accomplished using two-way analysis of variance (ANOVA) followed by Tukey’s honest significant difference (HSD). p-values < 0.05 were considered statistically significant.

## Results

### Phenotypic Characterization of DPSCs and PDLSCs

**Figure 1:**
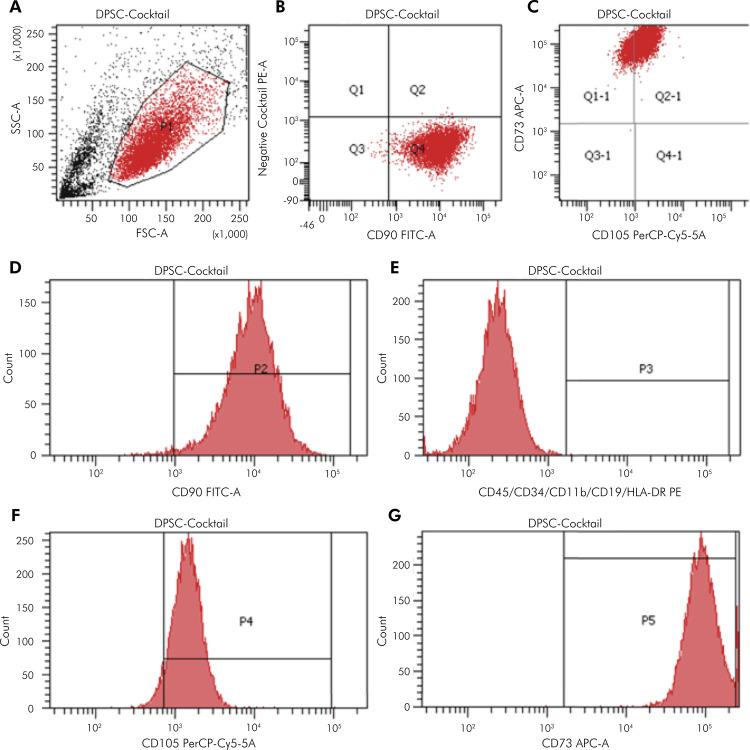
Flow cytometry results of DPSCs. DPSCs were harvested and labeled with specific antibodies for MSC markers as described in the Methodology. (A) Granularity and size of DPSCs. (B) A dot plot for a negative cocktail (CD45, CD34, CD11b, CD19, and HLA-DR) and CD90 antibody. (C) Dot plot for CD73 and CD105 antibodies. (D) Histogram for CD90. (E) Histogram for the negative cocktail. (F) Histogram for CD105. (G) Histogram for CD73.

APC-A: allophycocyanin area; FITC-A: fluorescein isothiocyanate area; FSC-A: forward scatter area; SSC-A: side scatter area; PE-A: phycoerythrin area; PerCP-Cy5-5-A: peridinin-chlorophyll-protein-cyanin 5.5 area.

**Figure 2:**
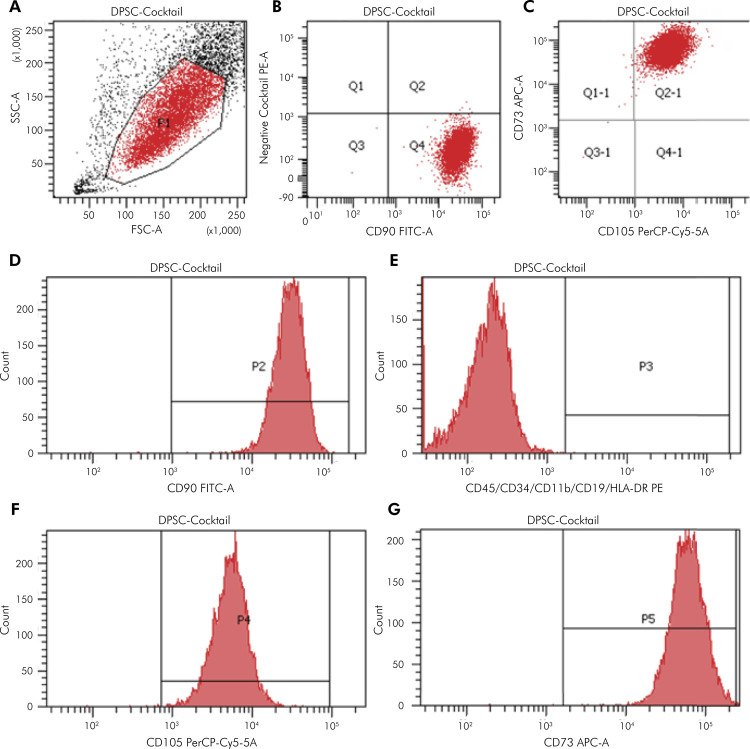
Flow cytometry results of PDLSCs. PDLSCs were harvested and labeled with specific antibodies for MSC markers as described in the Methodology. (A) Granularity and size of PDLSCs. (B) A dot plot for a negative cocktail (CD45, CD34, CD11b, CD19 and HLA-DR) and CD90 antibody. (C) Dot plot for CD73 and CD105 antibody. (D) Histogram for CD90. (E) Histogram for the negative cocktail. (F) Histogram for CD105. (G) Histogram for CD73.

APC-A: allophycocyanin area; FITC-A: fluorescein isothiocyanate area; FSC-A: forward scatter area; SSC-A: side scatter area; PE-A: phycoerythrin area; PerCP-Cy5-5-A: peridinin-chlorophyll-protein-cyanin5.5 area.

High expression of CD90, CD105, and CD73 (>95%) was exhibited by DPSCs and PDLSCs, whereas expression of negative markers were < 2% (Figures 1 and 2). The characteristics of these surface biomarkers matched the standard criteria defining MSCs proposed by the ISCT, suggesting that the cultured DPSCs and PDLSCs had the properties of MSCs.

### LPS Inhibited Osteogenic Differentiation of DPSCs and PDLSCs

**Figure 3:**
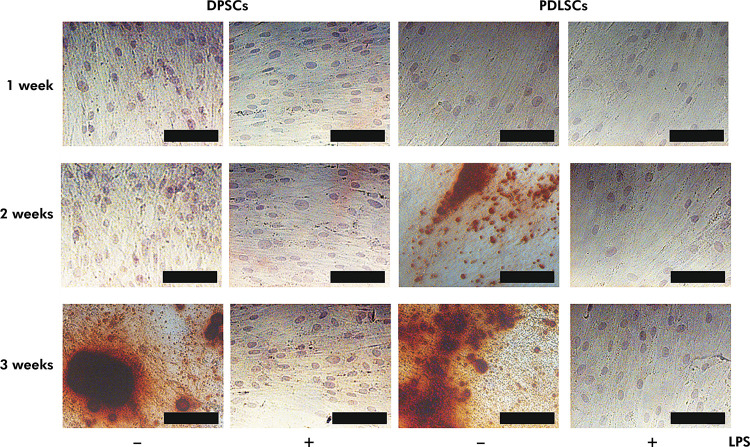
LPS inhibited osteogenic differentiation of DPSCs and PDLSCs. DPSCs and PDLSCs were cultured in an osteogenic medium and treated with/without LPS for 1, 2, or 3 weeks. DPSCs and PDLSCs were stained with alizarin red as described in the Methodology. Black bar: 100 µm.

Bone nodules, in the form of alizarin positive-red mineralized deposits, were observed in DPSCs on the third-week culture and in PDLSCs on the second-week culture under an inverted light microscope. No bone nodules were observed in 10 µg/mL LPS-supplemented DPSCs and PDLSCs after 1, 2, and 3 weeks (Figure 3).

### LPS-Induced NF-κB Activity in DPSCs and PDLSCs

**Figure 4:**
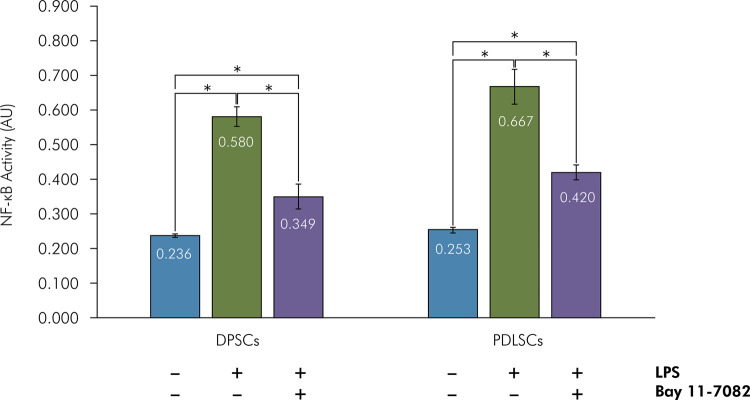
LPS induced NF-κB activity in DPSCs and PDLSCs. DPSCs and PDLSCs were cultured in an osteogenic medium, pretreated with/without 100 µM Bay 11-7082 for 30 min, and treated with/without 10 µg/mL LPS for 3 weeks. NF-κB activity was measured as described in the Methodology. The data are expressed as mean ± standard deviation (n = 6). *p < 0.05, Tukey’s HSD.

NF-κB activities of untreated DPSCs and PDLSCs were 0.236 ± 0.005 AU and 0.253 ± 0.008 AU, respectively. Following three weeks of LPS supplementation, NF-κB activities of DPSCs and PDLSCs were 0.580 ± 0.029 AU and 0.667 ± 0.051 AU. NF-κB activities of LPS-supplemented DPSCs and PDLSCs following pretreatment with Bay 11-7082 were 0.349 ± 0.037 and 0.420 ± 0.022 AU (Figure 4).

No significant interaction between the types of stem cells and treatments on NF-κB activity was indicated by two-way ANOVA (p = 0.148). NF-κB activity significantly differed in different treatment groups (p = 0.000). The 3-week-LPS-supplemented NF-κB activities of DPSCs and PDLSCs were significantly higher than those of untreated DPSCs and PDLSCs (p = 0.000) as well as those of Bay 11-7082-pretreated LPS-supplemented DPSCs and PDLSCs (p = 0.000). The NF-κB activities of untreated DPSCs and PDLSCs were significantly lower than those of Bay 11-7082-pretreated LPS-supplemented DPSCs and PDLSCs (p = 0.000). These results demonstrated that LPS-induced NF-κB activation in DPSCs and PDLSCs, and that Bay 11-7082 partially inhibited the LPS-induced NF-κB pathway.

### LPS Reduced ALP Activity and Inhibited Bone Nodule Formation in DPSCs and PDLSCs

**Figure 5:**
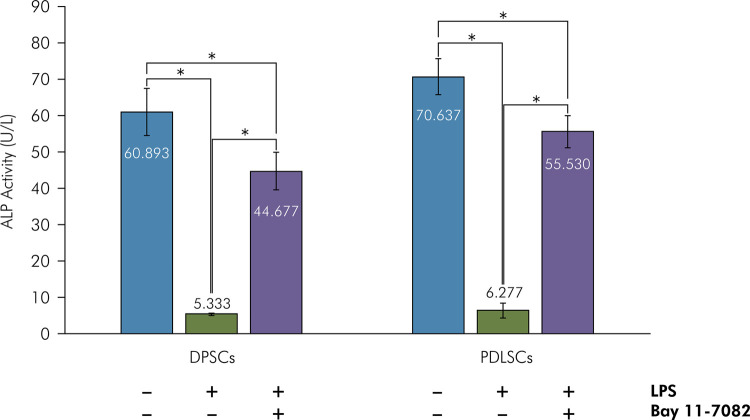
Bay 11-7082 prevented LPS-decreased ALP activity of DPSCs and PDLSCs. DPSCs and PDLSCs were cultured in an osteogenic medium, pretreated with/without 100 µM Bay 11-7082 for 30 min, and treated with/without 10 µg/mL LPS for 3 weeks. ALP activity was measured as described in the Methodology. The data are expressed as mean ± standard deviation (n = 6). *p < 0.05, Tukey’s HSD.

**Figure 6:**
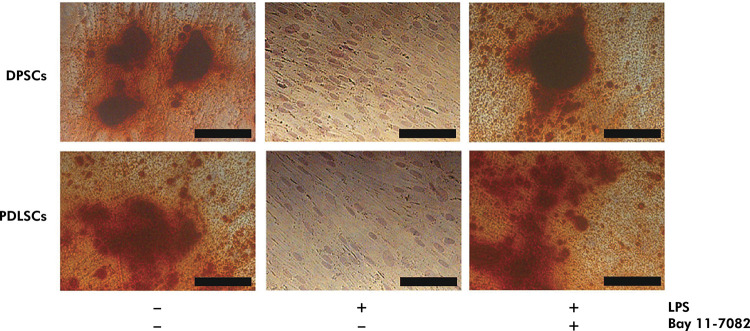
Bay 11-7082 prevented LPS-inhibited osteogenic differentiation of DPSCs and PDLSCs. DPSCs and PDLSCs were cultured in an osteogenic medium, pretreated with/without 100 µM Bay 11-7082 for 30 min, and treated with/without 10 µg/mL LPS and for 3 weeks. DPSCs and PDLSCs were stained with alizarin red as described in the Methodology. Black bar: 100 µm.

Two-way ANOVA did not indicate a significant interaction between stem cells and treatments on ALP activity (p = 0.148). Significant differences in ALP activity were observed in different treatment groups (p = 0.000). ALP activities of untreated DPSCs and PDLSCs were 60.893 ± 6.516 U/mL and 70.637 ± 4.902 U/mL, respectively. The ALP activities of DPSCs (5.333 ± 0.323 U/mL) and PDLSCs (6.277 ± 2.026 U/mL) were significantly lower than those of untreated DPSCs and PDLSCs after three weeks of LPS supplementation (p = 0.000) (Figure 5). Lower ALP activity was associated with the absence of bone nodule formation in LPS-supplemented DPSCs and PDLSCs (Figure 6). Pretreatment with Bay 11-7082 resulted in significantly higher ALP activities of LPS-supplemented DPSCs (44.677 ± 5.193 U/mL) and PDLSCs (55.530 ± 4.478 U/mL) compared with those supplemented with LPS (p = 0.000), but significantly lower than those of untreated (p = 0.000). These results showed that Bay 11-7082 was responsible for the partial maintenance of ALP activity in DPSCs and PDLSCs (Figure 5). Moreover, pretreatment with Bay 11-7082 partially maintained the osteogenic potency of LPS-supplemented DPSCs and PDLSCs (Figure 6).

## Discussion

LPS-induced NF-κB activation, was reported to play an important role in inflammatory responses and bone loss in periodontitis.^12^ This study demonstrated that P. gingivalis-derived LPS not only induced NF-κB activity but also inhibited bone nodule formation in DPSCs and PDLSCs. These findings are consistent with a previously conducted study that demonstrated that LPS-induced NF-κB activity impaired the osteogenic potency of gingival-derived mesenchymal stem cells (GMSCs).^19^ LPS supplementation also inhibited osteogenic differentiation in dental follicle stem cells (DFSCs).^20^

The activated NF-κB targeted the κB site and inhibit Smad in regulating *Runx2*,^21^ thereby inhibiting ALP production.^22^ In this study, bone nodule formation was clearly observed after 3 weeks of culturing with DPSCs and PDLSCs. In addition, ALP activity, which was observed in the 3-week culture, was reduced by LPS supplementation. Thus, NF-κB activity, which was induced by LPS, could reduce ALP activity in DPSCs and PDLSCs, leading to inhibition of bone nodule formation. This finding corroborates a previous study that revealed that LPS-induced NF-κB activity downregulated ALP mRNA and protein expressions in GMSCs.^19^ Furthermore, ALP activity was reported to be reduced by LPS in DFSCs.^20^

NF-κB signaling can be blocked by several substances and natural products,^23,24^ one of which is Bay 11-7082, which inhibits NF-κB activity in various types of stem cells, including BMMSCs,^25,26^ AdMSCs,^26^ and neural stem cells (NSCs).^27^ This study highlighted the role of Bay 11-7082 and its mechanism in maintaining osteogenic differentiation in LPS-stimulated DPSCs and PDLSCs. Bay 11-7082 supplementation led to the suppression of NF-κB activity, which was partially responsible for maintaining ALP activity and osteogenic potency in DPSCs and PDLSCs.

LPS could induce an inflammatory signaling pathway via NF-κB and other molecules, such as AP-1.^28^ Therefore, Bay 11-7082 was only able to partially suppress the inflammatory signaling pathway via NF-κB; however, AP-1 could still inhibit the osteogenic differentiation of DPSCs and PDLSCs. Consequently, further investigation of other inhibitors is necessary to enable complete suppression of the LPS-induced inflammatory signaling pathway, so that osteogenic differentiation of DPSCs and PDLSCs could be undisrupted.

## Conclusion

Inhibition of LPS-induced NF-κB activity can maintain the osteogenic potency of DPSCs and PDLSCs.
